# The Pronunciation of “U”

**Published:** 1882-06

**Authors:** 


### THE PRONUNCIATION OF "U."

Ninety-nine out of every hundred Northerners will say institoot instead of institute, dooty for duty—a perfect rhyme to the word beauty. They will call new and news noo and noos—and so on through the dozens and hundreds of similar words. Not a dictionary in the English language authorizes this. In student and stupid, the "u" has the same sound as in cupid, and should not be pronounced stoodent and stoopid, as so many teachers are in the habit of sounding them.

It is a vulgarism to call a door a doah—as we all admit—isn't it as much of a vulgarism to call a newspaper a noospaper? One vulgarism is Northern, and the other Southern, that's the only difference. When the *London Punch* wishes to burlesque the pronunciation of servants, it makes them call the duke the dook, the tutor the tooter, and a tube a toob. You never find the best Northern speakers, such as Wendell Philips, George William Curtis, Emerson, Holmes, and men of that class, saying noo for new, Toosday for Tuesday, avenoo for avenue, or calling a dupe a doop. It is a fault that a Southerner never falls into. He has slips enough of another kind, but he doesn't slip on the long "u." As many of our teachers have never had their attention called to this, I hope they will excuse this notice.—*Southern Letter.*

---

Look at This and Then at That.—Miss Blanche Murray is a very proper young lady. Last week she caught her little brother smoking.

"You terrible thing," she hissed, "I am going to tell father."

"This is only corn silk," murmured the boy, penitently.

"I don't care what it is. I am going to tell on you, and see that you don't get into that beastly, horrid, degrading habit."

II.

It is evening. Miss Murray is sitting on the front stoop with Algernon. It is moonlight, and the redolent spirits of the honeysuckles and syringa are wafting bliss to their intoxicated souls.

"Would little birdie object to my smoking a cigarette?"

"Not at all," replied Miss Murray. "I like cigarettes. They are so fragrant and romantic. I think they are just too delicate for anything."

"Then I'll light one."

He lights a cigarette, and they talk about the weather for two hours and a half.—*Petroleum World.*

